# Post-discharge digital self-management for acute coronary syndrome patients: A scenario-based design and implementation

**DOI:** 10.1007/s12471-025-01993-w

**Published:** 2025-10-15

**Authors:** Annemiek Vredenburg-Jimmink, Dafne Umans, Stijn Wierdsma, Marianne Schoenmakers, Victor Umans

**Affiliations:** 1 Department of Cardiology, Noordwest Ziekenhuisgroep, Alkmaar, The Netherlands; 2 Department of Emergency Medicine, Noordwest Ziekenhuisgroep, Alkmaar, The Netherlands; 3 Noordwest Ziekenhuisgroep Alkmaar, Alkmaar, The Netherlands

## Introduction

In 2023, 33,600 people developed a myocardial infarction in the Netherlands [1], and their PCIs for STEMI or NSTEMI are registered annually in the NHR registry [2].

Despite advances in hospital-based ACS treatment, the post-discharge phase remains a high-risk period characterized by poor medication adherence, lifestyle regression, fragmented care coordination, and unexpected readmissions [3, 4]. Improvement of appropriate care may be enhanced by improving patient self-management and increasing disease knowledge. Cardiac rehabilitation programs are underutilized, and many patients may be supported by digital health techniques, which should be designed with their needs, health literacy, and clinical workflows in mind [5].

The introduction of IT modalities may help clinicians overcome these care gaps. These new solutions should be proven to be safe to use in an outpatient setting as a monitoring option and may become a potential modality to help patients manage their disease.

Therefore, we modeled an innovative IT-guided ACS connected care system with the aim of following patients after their recent myocardial infarction. Secondly, we studied its 6‑week impact on: patient education and satisfaction, home monitoring of blood pressure and reduction of (un)scheduled outpatient visits.

## Methods

### Design approach

We used a scenario-based design approach [6] to model the desired functionalities and user experience. The scenario was developed using qualitative input from a patient panel, nurse practitioners, cardiologists, the coronary care unit, and rehabilitation nurses. Functional requirements were derived from four recurring themes: patient confusion, low self-efficacy, care discontinuity, and lack of remote insight into patient recovery. Fundamentally, the new design also incorporates a new outpatient follow-up scheme, omitting the regular 2‑week lab value controls and the scheduled 4‑week outpatient ACS clinic visit.

### The Walt scenario

“Walt,” a 75-year-old male STEMI patient who underwent a primary percutaneous coronary intervention served as the user archetype. After discharge, the ACS app needed to improve patients’ like “Walt” insight into their condition even after treatment. This new modality should incorporate items to help patients realize the impact of their risk factors and how behavioral changes play a role. His digital journey from hospitalization through disease-education, rehabilitation and home-based self-management was scripted to illustrate critical system interactions. Features and workflows were prototyped at high fidelity and fitted into the new innovative IT-modality including a messenger service for educational push messages. At onboarding, the CCU nurse installs the ACS app on the patient’s own smartphone and guides the patient and family through the app’s components, offering continuity and clarity [5]. Coaching interventions include activity challenges and motivational messaging based on trends.

## Results

### Patient stratification and onboarding

**Fig. 1:**
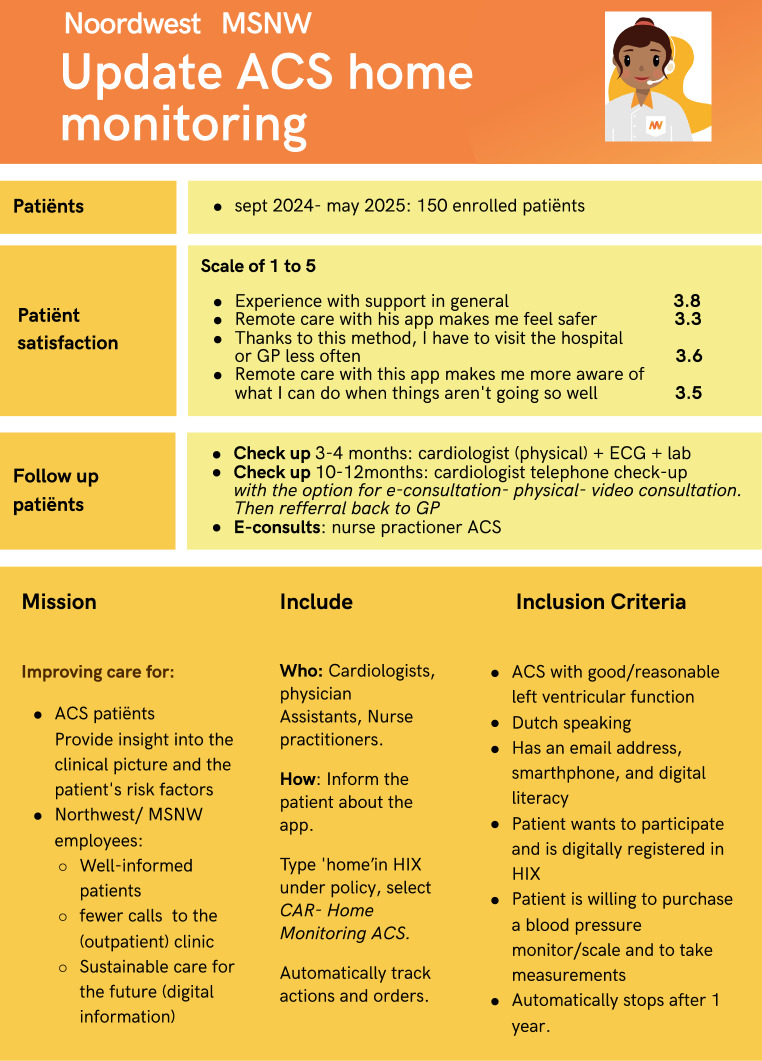
Outline of the Noordwest home monitoring application for ACS patients

Figure 1 shows the implementation and outcome in 150 NSTEMI/STEMI patients (77% males, 63 (45–85)) years. All eligible patients with moderate and good left ventricular function were stratified on day one at the CCU during the clinical rounds. A nurse initiates the novel ACS care plan, incorporating lab orders, intervention summaries, behavior goals, and medication.

### Monitoring and engagement tools

All consented patients had their app activated and were monitored for 365 days. The app tracks blood pressure, heart rate, weight, symptoms, and activity as reported by the patient. The app is connected to the electronic health record, and all measurements are presented in that environment. Patients provided their weight, blood pressure, and heart rate every 14 days, and their number of steps every month.

### Tailored education and coaching

Educational videos and tips were delivered weekly via a newsfeed, matching the patients recovery phase and risk profile. All patients received a message on each working day during the first 2 months. Initially, the focus was on educational information, but in the subsequent months, the messages became more focused on lifestyle changes/adherence and contain motivational tools. Active smokers received every 28 days a quit smoking question/advice.

### Remote telemonitoring and reduction in (un)planned visits

In all cases, nurses of the NWZ monitoring center were able to monitor patient dashboards, chat with patients, respond to questions, and push custom content or follow-up questionnaires. Only unexpected out-of-range laboratory values were forwarded through the monitoring center nurses to the attending nurse practitioner.

Audiovisual consults replaced physical visits when needed. Individual trends were used to plan follow-ups and inform medication changes or interventions. The attending cardiologist was available for symptom interpretation and clinical decision-making. Thus, the first outpatient visit within 30 days was replaced by this telemonitoring concept in all but 8 (5%) patients, and none of the patients presented to the emergency department.

## Discussion

This digital care framework addresses known barriers in ACS recovery: patient disorientation post-discharge, lack of between-visit monitoring, and limited access to cardiac rehab [4, 6, 7]. This approach offers a feasible pathway for blending education, tracking, and clinician oversight. Patients feel empowered and have fewer urgencies to seek clinical support at the outpatient clinic or by telephone. The implementation of the app reduced the 30-day outpatient visits by 95% with a patient satisfaction rated at 3.6 out of 5.

By using data-driven personalization and increasing patient awareness, the system may encourage adherence and enable early identification of deterioration. The integration of FAQ answers and symptom notes empowers patients without overwhelming them. Thus, healthcare consumption may be shifted to remote contact only when patients are in need, thereby abandoning routine appointments in the first 3 months after ACS [7]. Nationally, such innovative approaches could result in a reduction of up to 95% of outpatient visits within 6 weeks, combined with an accompanying reduction in CO2 emissions [8].

Thus, this system offers a promising model for bridging the post-discharge care gap for ACS patients. Future clinical validation and further integration with electronic health records are needed to assess efficacy and scalability.

## Limitations

This single-center experience has some caveats: patients need to have a smartphone and have and adequate cognitive abilities to use smartphone apps. Only patients with moderate and good left ventricular function were enrolled.
